# A multicenter study of the prevalence and risk factors of malaria and anemia among pregnant women at first antenatal care visit in Ghana

**DOI:** 10.1371/journal.pone.0238077

**Published:** 2020-08-21

**Authors:** Linda Ahenkorah Fondjo, Otchere Addai‑Mensah, Max Efui Annani-Akollor, Jude Tetteh Quarshie, Adwoa Abrafi Boateng, Samuel Ernest Assafuah, Eddie-Williams Owiredu

**Affiliations:** 1 Department of Molecular Medicine, School of Medicine and Dentistry, College of Health Sciences, Kwame Nkrumah University of Science and Technology, Kumasi, Ghana; 2 Department of Medical Diagnostics, Faculty of Allied Health Sciences, College of Health Sciences, Kwame Nkrumah University of Science and Technology, Kumasi, Ghana; Instituto Rene Rachou, BRAZIL

## Abstract

**Background:**

Malaria in pregnancy remains a major public health problem in Africa and Ghana and has been associated with a variety of pregnancy-related adverse complications. The development of effective and timely health policies for the prevention and control of malaria and anemia in pregnancy; requires current and consistent data on the prevalence and risk factors. We report the prevalence and risk factors of malaria and anemia from three major hospitals across three regions in Ghana.

**Methods:**

This multicenter cross-sectional study comprising a total of 628 pregnant women was conducted at the antenatal care units of the Achimota Hospital in the Greater Accra Region (n = 199), St. Michael’s Hospital in the Ashanti Region (n = 221), and Effia Nkwanta Regional Hospital in the Western Region (n = 211). Questionnaires were administered to obtain socio-demographic, obstetrics and clinical data. Venous blood, stool and urine samples were collected for hematological profile and parasite identification using microscopy. Risk factors were evaluated using logistic regression models.

**Results:**

The overall prevalence of *P*. *falciparum* malaria was 8.9%. Factors independently associated with malaria were self-reported mosquito exposure (moderate exposure: aOR = 3.11, 95% CI (1.12–8.61) and severe exposure: aOR = 10.46, 95% CI (3.86–28.34)) and non-use mosquito repellents (aOR = 3.29, 95% CI (1.70–6.39)). Multiparty (parity of 2: aOR = 0.19, 95% CI (0.05–0.70) and parity ≥3: aOR = 0.11, 95% CI (0.03–0.45)) and age (20–30 years old: aOR = 0.22, 95% CI (0.09–0.56)) reduced the odds of infection. The overall prevalence of anemia was 42.4%. The prevalence of mild, moderate and severe anemia were 35.7%, 6.1% and 0.6%, respectively. The use of water other than purified water (tap water: aOR = 3.05, 95% CI (2.06–4.51) and well water: aOR = 2.45, 95% CI (1.35–4.44)), increasing gestational age (second trimester: aOR = 2.05, 95% CI (1.41–2.97) and third trimester: aOR = 7.20, 95% CI (3.06–16.92)) and malaria (aOR = 2.40, 95% CI (1.27–4.53)) were independent risk factors for anemia.

**Conclusions:**

Although the prevalence of malaria is relatively low, that of anemia remains high. We recommend increasing efforts to make ITNs more available to strengthen malaria prevention. Public health education programs could help improve uptake and proper use of ITNs. To help reduce anemia in pregnancy, women should be empowered economically and interventions that reduce malnutrition should be encouraged. Women should be educated on early initiation of antenatal care to enhance surveillance, identification and treatment of anemia.

## Introduction

Malaria is a parasitic disease, mostly prevalent in sub-Saharan Africa, Asia, and Latin America [1]. Data from the World Health Organization (WHO) indicate about 219 million cases and 435,000 malaria-related deaths globally [2]. In the WHO African Region, malaria causes significant morbidity and mortality with annual infection and mortality rates of 213 million and 380,000 individuals, respectively [3, 4]. There have been some successes in the global control of malaria; however, current evidence suggests insufficient progress, particularly in the African context [1]. In Ghana, malaria is still a major cause of loss of days of healthy life and accounts for more than 20% of child deaths, 40% of child hospital admissions, and more than 50% of outpatient attendances [5, 6].

Malaria infection during pregnancy is indeed a major public health problem in tropical and subtropical regions with an estimated 24 million pregnant women being infected in sub-Saharan Africa each year [7]. The risk of infection and morbidity is high in primiparous women, adolescents, and those co-infected with human immunodeficiency virus (HIV); and malaria-related maternal deaths each year is estimated at over 25% in endemic areas [8]. Malaria during pregnancy may cause a variety of adverse complications including placental malaria, low birth weight from prematurity and intrauterine growth retardation, congenital infection and infant mortality [9]. Another critical sequela of malaria in pregnancy is maternal anemia.

Anemia in pregnancy is an important global public health problem. The WHO defines anemia in pregnancy as having haemoglobin (Hb) concentration of less than 11 g/dL [10]. It is estimated that more than 40% of pregnant women worldwide are anemic and it is considered a severe public health problem if the anemia is present in ≥40% of the population [10]. In Africa, anemia in pregnancy affects approximately 50% of pregnant women in malaria-endemic countries [11]. The etiology of anemia is multifactorial, with nutritional deficiencies (iron and folate), infectious diseases (hookworm, schistosomiasis, malaria and HIV) and genetic red blood cell disorders (sickle cell and thalassaemias) being key contributors [12]. Other factors such as socio-demographic and economic factors such as place of residence and educational level also play important roles [13]. The relationship between malaria and anemia is in the role played by malaria in the depletion of non-parasitized erythrocytes, immune destruction of parasitized red cells, and impaired erythropoiesis as a result of bone marrow dysfunction [14–16]. Malaria accounts for an estimated 3–15% of anemia and 25% of severe anemia in pregnant women from malaria-endemic countries [9]. Anemia in pregnancy is also a contributing factor for maternal death, still births, low birth weights, and impairment of fetal development [17].

The detrimental effect of malaria and anemia on the mother and fetus underscore the need for more aggressive and sustainable preventive and therapeutic measures. The WHO recommends sleeping under insecticide-treated nets (ITNs) to reduce contact between mosquitoes and humans and use of intermittent preventive treatment (IPTp) with sulphadoxine-pyrimethamine (SP) at each scheduled antenatal visit after the first trimester [1]. ITNs have been shown to reduce maternal malaria parasitemia by 38%, malaria-related anemia by 47%, low birth weight by 23%, miscarriages/stillbirths by 33% and placental parasitemia by 23% [18, 19]. Notwithstanding the importance of the WHO recommendations, recent evidence in Ghana suggest that compliance to the use of ITN and IPTp-SP remains low [20–22].

There is thus the need for effort intensification to ensure compliance and reduce malaria and anemia in pregnancy. This would require the availability of reliable statistics on the prevalence and risk factors of malaria and anemia in pregnancy across the country. Although some cross-sectional studies have been conducted in Ghana [23–26], the development of effective and timely health policies for prevention and control of malaria and anemia in pregnancy and its associated adverse complications requires current and consistent data on the prevalence and risk factors. Here, we report the prevalence and risk factors of malaria and anemia from three major hospitals across three regions in Ghana.

## Materials and methods

### Study design/setting

**Fig 1:**
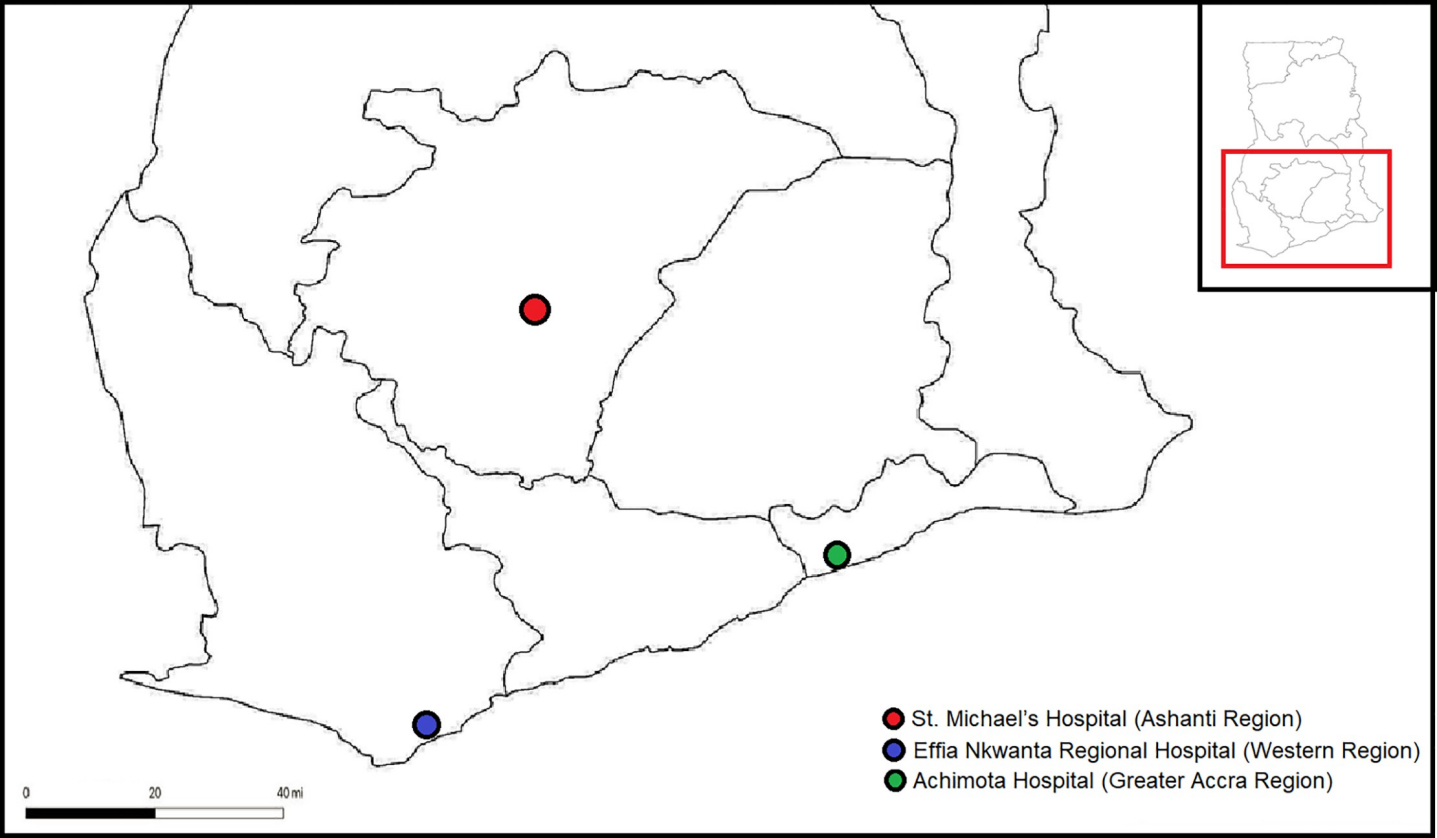
Map showing the locations of the study area.

This multicenter cross-sectional study was conducted from July to August 2018 at the antenatal care (ANC) units of conveniently sampled three major hospitals in three different regions of Ghana: Achimota Hospital in the Greater Accra Region, St. Michael’s Hospital in the Ashanti Region, and Effia Nkwanta Regional Hospital in the Western Region. The Achimota Hospital is located in the Accra Metropolitan District, a district in the Greater Accra Region of Ghana. As a part of Accra, Achimota experiences a bi-modal rainy season: April through June and September through November. It is within these times of the year that malaria transmission is highest. St. Michael’s Hospital is located at Pramso in the Bosomtwe District of the Ashanti Region of Ghana. The Ashanti Region has an intense perennial malaria transmission, with the predominant parasite being *P*. *falciparum*. Effia Nkwanta Regional Hospital is located in the Sekondi-Takoradi Metropolis, the capital of the Western Region of Ghana. The Metropolis has a total land area of 192 km^2^; an average annual growth rate of 3.2% and the prevalence of malaria in pregnancy in the region was 26.1% in 2015 [27]. The locations of the study area are indicated in Fig 1.

### Study population

**Fig 2:**
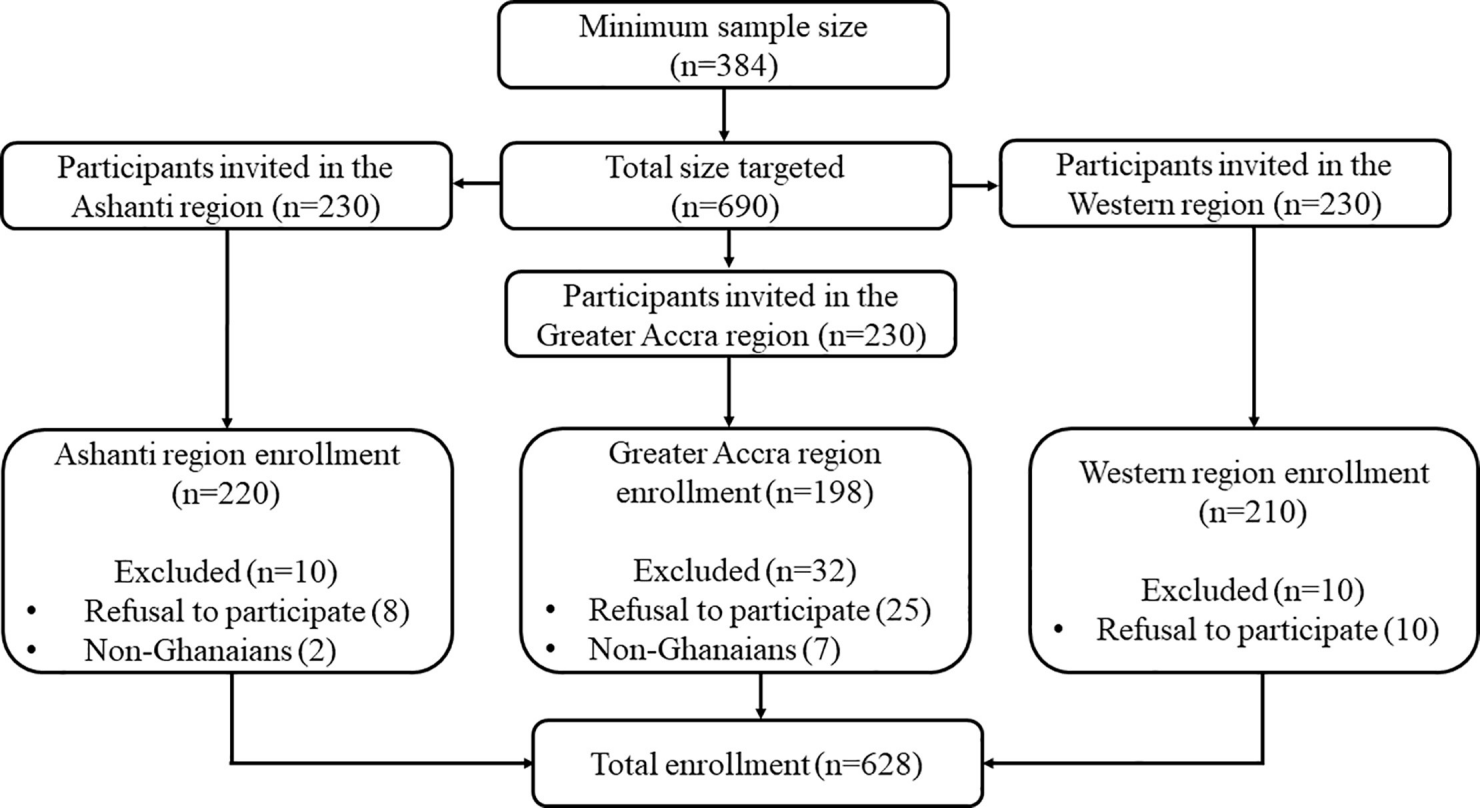
Flowchart showing selection of study participants.

The MedCalc Statistical Software version 18.9.1 (MedCalc Software bvba, Ostend, Belgium) was used to calculate the sample size for this study. Based on the estimated population of Ghana (30 million), a 50.0% response distribution, 95% confidence level, 5% margin of error, a study power of 80%, and design effect of 1, the minimum sample size required for this study was 384. However, in an effort to strengthen statistical power, a total of 628 consecutive consenting pregnant women (comprising 198 from the Achimota Hospital, 220 from the St. Michael’s Hospital and 210 from the Effia Nkwanta Regional Hospital) attending their first ANC visit and who have been resident in the study area for at least 6 months were recruited for the study. Excluded participants were non-Ghanaian pregnant women and those who were in critical condition and needed emergency care. We included only Ghanaians to limit the recruitment of non-Ghanaians who were within the region for only a short time. All the non-Ghanaians we encountered during sampling had only been in the country for a short duration (<6 months) and thus, were all excluded. All pregnant women who met the inclusion criteria and consented after the aim and objectives had been explained to them were eligible to participate in the study. Fig 2 shows the flowchart for participants’ selection.

### Ethical considerations

This study was approved by the Committee for Human Research and Publication Ethics (CHRPE), of the School of Medical Sciences, Kwame Nkrumah University of Science and Technology (CHRPE/AP/137/19). Participation was fully voluntary and written consent was obtained from each participant.

### Questionnaire administration

To each participant, a validated semi-structured questionnaire, designed by reviewing previous studies of similar objectives and modifying to suit our study objective, was administered to obtain socio-demographic and lifestyle characteristics data (age, marital status, educational level, employment status, home type, availability of electricity, source of drinking water, self-reported mosquito exposure, ITN use and treatment, repellent use and type of repellent); obstetrics and clinical data. Validation of the questionnaire was performed on 30 participants. The internal consistency, evaluated based on Cronbach’s alpha, was 0.72. Details of the questionnaire can be found in S1 File (Questionnaire).

### Hemodynamic and anthropometric measurements

Blood pressure was measured with an automated blood pressure apparatus (Omron MX3-Omron Matsusaka Co., Ltd. Japan) from the right arm after the study participant had been made to sit for at least five minutes. The average of the two readings taken five minutes apart was recorded. Height was measured without wearing shoes using a wall mounted ruler, to the nearest 0.1 m. Waist and hip circumferences were measured to nearest centimeter using a measuring tape. Waist to hip ratio (WHR) = WC (m)/HC (m), waist to height ratio and (WHtR) = WC (m)/height (m) were calculated. Body mass index (BMI) was calculated using the equation; [BMI (kg/m2) = weight/height^2^].

### Laboratory investigations

Five milliliters (5 ml) of venous blood sample was collected from each participant and dispensed in K_3_ EDTA tubes for full blood count (FBC) and malaria parasite detection. FBC was performed using the Mindray BC-3000Plus Auto Haematology Analyzer. Ten percent Giemsa-stained thick and thin films were prepared on clean grease-free slides (thin films were fixed with methanol) for malaria microscopy. Examination and reporting of both thick and thin films were performed independently by two trained microscopists. Thick films were used for malaria parasite count and thin films were used to identify the specific species of *Plasmodium*. A film was considered positive when both microscopists recorded a positive result and for the same species. A film was considered negative only after observing at least 200 high-power fields (HPF) without finding parasites on a thick film. In the case of discordant results, a third microscopist was employed to break the tie. The tests were performed immediately after sample collection to minimize storage variabilities. Additionally, to each participant, two well-labelled, wide-mouth and screw-capped containers were given and instructed to bring their early morning stool and urine samples the following day. The stool and urine samples were immediately transported in cold boxes at 4°C to the laboratory department of each hospital for processing and examination. Urine and stool routine examination were done for each urine and stool sample, respectively. For all stool and urine parasitological tests, samples were considered as positive if the eggs/ova/cysts/trophozoites were detected. For quality control purposes, all slides were prepared and examined in duplicates.

### Definition for anemia

Anemia was defined as hemoglobin (Hb) level < 11g/dl; mild anemia was defined as 9g/dl ≤ Hb < 11g/dl; moderate anemia was defined as 7g/dl ≤ Hb < 9g/dl and severe anemia was defined as Hb < 7g/dl [28].

### Data analysis

Statistical analysis was performed using the R Language for Statistical Computing version 3.6.0 [29]. Categorical data were presented as frequencies (percentages). Continuous data were presented as mean ±SD or median (interquartile ranges), where appropriate. To determine potential factors associated with malaria or anemia, we first performed Chi squared and Fisher exact tests where applicable, after which statistically significant variables were selected for collinearity assessment using the variance inflation factor (VIF) (VIF for all significant variables after univariate analyses were <5), followed by multivariate logistic regression analysis, using the enter method, to identify independent risk factors with adjustment for study site. Multivariate regression analysis for anemia was further adjusted for the presence of urine blood, urine protein and use of medication (hematinic, folic acid and herbal medication). All tests were two-sided and p-value < 0.05 was considered statistically significant.

## Results

A total of 628 pregnant women with mean age of 28.44±6.19 years participated in the study. Most of the participants were married (454/72.3%), had secondary education (416/66.2%), were employed (516/82.2%) and a total of 166 (26.4%) self-reported having been severely exposed to mosquito bites within the past month. Less than half of the participants (282/45.2%) used ITN and of this, only 2 (0.3%) treat their ITN. Notwithstanding, a higher proportion of the participants used mosquito repellents (468/74.5%), with mosquito insecticide spray being the most commonly used (324/69.2%) (Table 1).

**Table 1:** Sociodemographic and lifestyle characteristics of the study population.

Variables	Frequencies (n = 628)	Percentages (%)
Age (years)*	28.44±6.19	
<20	60	9.6
20–30	334	53.2
>30	234	37.3
**Marital status**		
Single**	174	27.7
Married	454	72.3
**Educational level**		
No formal education	38	6.1
Primary	82	13.1
Secondary	416	66.2
Tertiary	92	14.6
**Employment status**		
Unemployed	112	17.8
Employed	516	82.2
**Home type**		
Self-owned house	310	49.4
Compound/shared house	318	50.6
**Electricity**		
Absent	50	8.0
Present	578	92.0
**Source of drinking water**		
Purified water	332	52.9
Tap water	232	36.9
Well water	64	10.2
**Self-reported mosquito exposure**		
Barely	220	35.0
Moderately	242	38.5
Severely	166	26.4
**ITN use**		
No	334	54.8
Yes	284	45.2
*ITN treatment*	*2*	*0*.*3*
**Repellent use**		
No	160	25.5
Yes	468	74.5
*Spray*	*324*	*69*.*2*
*Mosquito coil*	*240*	*51*.*3*

*Mean±SD

**Single: (not married, divorced or widowed); ITN: insecticide treated net

The obstetrics and clinical characteristics of the participants is shown in Table 2. The mean gestational age for the entire study population was 3.63±1.41 months. Most of the participants were in their second trimester (336/53.5%) and were nulliparous (194/30.9%). Hundred and twelve (17.8%) and 20 (3.2%) participants presented with proteinuria and hematuria, respectively. However, none of the participants had *Schistosoma haematobium* infection upon urine microscopy. Stool examinations were negative for both intestinal flagellates and helminths among all participants (Table 2).

**Table 2:** Obstetrics and clinical characteristics.

Variables	Frequencies (n)	Percentages (%)
**Obstetric parameters**		
Gestational age (months)*	3.63±1.41	
First trimester	256	40.8
Second trimester	336	53.5
Third trimester	36	5.7
Parity		
0	194	30.9
1	160	25.5
2	112	17.8
≥3	162	25.8
Body temperature (°C)*	36.36±0.62	
**Hemodynamic and anthropometry**		
SBP (mmHg)*	110.41±11.17	
DBP (mmHg)*	69.08±8.92	
BMI (kg/m^2^)*	25.91±5.10	
WHR*	0.89±0.06	
WHtR*	0.57±0.08	
**Hematological parameters**		
RBC (x10^6^/μL)*	4.05±0.61	
Hemoglobin (g/dL)*	11.11±1.43	
HCT (%)*	34.41±6.68	
MCV (fL)*	85.11±11.58	
MCH (pg)*	27.80±4.25	
MCHC (g/dL)*	32.91±3.95	
PLT (x10^3^/μL)‡	234.00 (191.00–304.00)	
WBC (x10^3^/μL)*	7.79±3.01	
**Urine assessment**		
Urine protein	112	17.8
Urine blood	20	3.2
*Schistosoma haematobium*	0	0.0
**Stool assessment**		
Intestinal flagellate	0	0.0
Helminths	0	0.0
**Treatments**		
Hematinics	144	22.9
Folic acid	98	15.6
Herbal medicines	32	5.1
Antipyretics	32	5.1

*Mean±SD

‡; median (interquartile range); SBP: systolic blood pressure; DBP: diastolic blood pressure; BMI: body mass index; WHR: waist-to-hip ratio; WHtR; waist-to-height ratio; RBC: red blood cell count; HCT: hematocrit; MCV: mean cell volume; MCH: mean cell hemoglobin; MCHC: mean cell hemoglobin concentration; PLT: platelet count; WBC: white blood cell count

**Fig 3:**
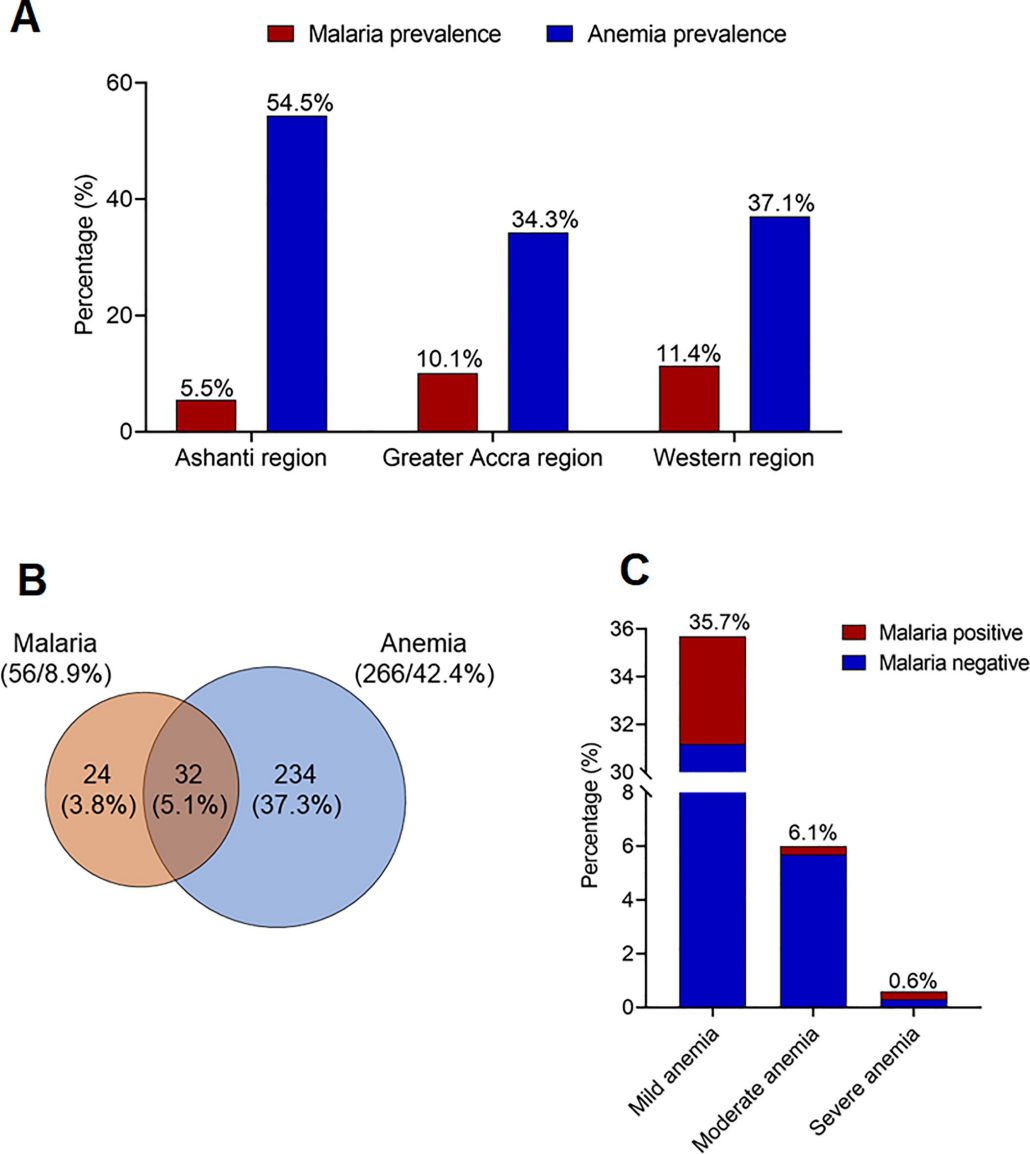
Prevalence of anemia and malaria among the study population. (A) Proportion of subjects with anemia, malaria and malaria-related anemia (B) Prevalence of the degrees of anemia by malaria status.

Only *Plasmodium falciparum* was detected in this study. The prevalence of *P*. *falciparum* malaria was 5.5%, 10.1% and 11.4% in the Ashanti, Greater Accra and Western regions, respectively (Fig 3A). The overall prevalence of malaria among the study population was 8.9% (Fig 3B). The prevalence of anemia was 54.5%, 34.3% and 37.1% in the Ashanti, Greater Accra and Western regions, respectively (Fig 3A). The overall prevalence of anemia was 42.4%, with 35.7%, 6.1% and 0.6% presenting with mild, moderate and severe anemia, respectively (Fig 3B and 3C). A total of 32 (5.1%) of the anemic participants comprising 4.5%, 0.3% and 0.3% with mild, moderate and severe anemia, respectively, also had malaria (Fig 3).

A higher number of the participants who had malaria were within 20–30 years old (24/7.2%), had secondary education (38/9.1%), were employed (48/9.3%), did not use ITN (42/12.2%), self-reported severe exposure to mosquito bites (32/19.3%), were in their first trimester of pregnancy (30/11.7%) and were nulliparous (32/16.5%). Univariate analysis revealed statistically significant associations between malaria and age, marital status, source of drinking water, self-reported mosquito exposure, parity, use of ITN and mosquito repellents (Table 3).

**Table 3:** Factors associated with malaria among the study population.

Variables	Plasmodium parasites		p-value
	Absent (n = 572)	Present (n = 56)	
**Age (years)**			<0.0001
<20	40 (66.7)	20 (33.3)	
20–30	310 (92.8)	24 (7.2)	
>30	222 (94.9)	12 (5.1)	
**Marital status**			<0.0001
Single	146 (83.9)	28 (16.1)	
Married	426 (93.8)	28 (6.2)	
**Educational level**			0.497
No formal education	36 (94.7)	2 (5.3)	
Primary	72 (87.8)	10 (12.2)	
Secondary	378 (90.9)	38 (9.1)	
Tertiary	86 (93.5)	6 (6.5)	
**Employment status**			0.584
Unemployed	104 (92.9)	8 (7.1)	
Employed	468 (90.7)	48 (9.3)	
**Home type**			0.263
Self-owned house	278 (89.7)	32 (10.3)	
Compound/shared house	294 (92.5)	24 (7.5)	
**Electricity**			0.073
Absent	42 (84.0)	8 (16.0)	
Present	530 (91.7)	48 (8.3)	
**Source of drinking water**			0.029
Purified water	298 (89.8)	34 (10.2)	
Tap water	210 (90.5)	22 (9.5)	
Well water	64 (100.0)	0 (0.0)	
**Self-reported mosquito exposure**			<0.0001
Barely	214 (97.3)	6 (2.7)	
Moderately	224 (92.6)	18 (7.4)	
Severely	134 (80.7)	32 (19.3)	
**ITN use**			0.002
No	302 (87.8)	42 (12.2)	
Yes	270 (95.1)	14 (4.9)	
**Repellent use**			<0.0001
No	134 (83.8)	26 (16.3)	
Yes	438 (93.6)	30 (6.4)	
**Obstetric parameters**			
**Gestational age**			0.118
First trimester	226 (88.3)	30 (11.7)	
Second trimester	312 (92.9)	24 (7.1)	
Third trimester	34 (94.4)	2 (5.6)	
**Parity**			<0.0001
0	162 (83.5)	32 (16.5)	
1	144 (90.0)	16 (10.0)	
2	108 (96.4)	4 (3.6)	
≥3	158 (97.5)	4 (2.5)	

ITN: insecticide treated net

Multivariate logistic regression analysis identified age (20–30 years old: aOR = 0.22, 95% CI (0.09–0.56), p = 0.001) and multiparty (parity of 2: aOR = 0.19, 95% CI (0.05–0.70), p = 0.013 and parity ≥3: aOR = 0.11, 95% CI (0.03–0.45), p = 0.002) to be independently associated with lower odds of malaria. Self-reported mosquito exposure (moderate exposure: aOR = 3.11, 95% CI (1.12–8.61), p = 0.029 and severe exposure: aOR = 10.46, 95% CI (3.86–28.34), p<0.0001) and non-use of mosquito repellents (aOR = 3.29, 95% CI (1.70–6.39), p<0.0001) were associated with increased odds of malaria infection (Table 4).

**Table 4:** Multivariate regression analysis for factors associated with malaria.

Variables	aOR (95%CI)	p-value
**Age (years)**		
<20	1	
20–30	0.22 (0.09–0.56)	0.001
>30	0.52 (0.17–1.69)	0.275
**Marital status**		
Single	1	
Married	0.87 (0.42–1.79)	0.699
**Source of drinking water**		
Purified water	1	
Tap water	0.81 (0.39–1.70)	0.578
Well water	na	na
**Self-reported mosquito exposure**		
Barely	1	
Moderately	3.11 (1.12–8.61)	0.029
Severely	10.46 (3.86–28.34)	<0.0001
**ITN use**		
Yes	1	
No	1.05 (0.49–2.22)	0.909
**Repellent use**		
Yes	1	
No	3.29 (1.70–6.39)	<0.0001
**Parity**		
0	1	
1	0.79 (0.36–1.73)	0.559
2	0.19 (0.05–0.70)	0.013
≥3	0.11 (0.03–0.45)	0.002

na: not applicable, ITN: insecticide treated net

Most of the participants who had anemia were 20–30 years old (140/41.9%), married (174/38.3%), had secondary education (172/41.3%), were employed (212/41.1%), used purified drinking water (104/31.3%), were in the second trimester (160/47.6%) and were nulliparous (94/48.5%). There were statistically significant associations between anemia and marital status, source of drinking water, gestational age, parity and malaria based on univariate analysis (Table 5).

**Table 5:** Factors associated with anemia among the study population.

Variables	Anemia		p-value
	Absent (n = 362)	Present (n = 266)	
**Age (years)**			0.051
<20	26 (43.3)	34 (56.7)	
20–30	194 (58.1)	140 (41.9)	
>30	142 (60.7)	92 (39.3)	
**Marital status**			0.001
Single	82 (47.1)	92 (52.9)	
Married	280 (61.7)	174 (38.3)	
**Educational level**			0.221
No formal education	16 (42.1)	22 (57.9)	
Primary	46 (56.1)	36 (43.9)	
Secondary	244 (58.7)	172 (41.3)	
Tertiary	56 (60.9)	36 (39.1)	
**Employment status**			0.172
Unemployed	58 (51.8)	54 (48.2)	
Employed	304 (58.9)	212 (41.1)	
**Home type**			0.872
Self-owned house	180 (58.1)	130 (41.9)	
Compound house	182 (57.2)	136 (42.8)	
**Source of drinking water**			<0.0001
Purified water	228 (68.7)	104 (31.3)	
Tap water	102 (44.0)	130 (56.0)	
Well water	32 (50.0)	32 (50.0)	
**Obstetric parameters**			
**Gestational age**			<0.0001
First trimester	176 (68.8)	80 (31.3)	
Second trimester	176 (52.4)	160 (47.6)	
Third trimester	10 (27.8)	26 (72.2)	
**Parity**			0.030
0	100 (51.5)	94 (48.5)	
1	106 (66.3)	54 (33.8)	
2	68 (60.7)	44 (39.3)	
≥3	88 (54.3)	74 (45.7)	
**Malaria**			0.023
Absent	338 (59.1)	234 (40.9)	
Present	24 (42.9)	32 (57.1)	

In multivariate analysis, use of water other than purified water (tap water: aOR = 3.05, 95% CI (2.06–4.51), p<0.0001 and well water: aOR = 2.45, 95% CI (1.35–4.44), p = 0.003), increasing gestational age (second trimester: aOR = 2.05, 95% CI (1.41–2.97), p<0.0001 and third trimester: aOR = 7.20, 95% CI (3.06–16.92), p<0.0001) and malaria (aOR = 2.40, 95% CI (1.27–4.53), p = 0.007) were independent risk factors for anemia. Being married was associated with lower odds of anemia (aOR = 0.64, 95% CI (0.42–0.99), p = 0.044) after controlling for the hematuria, proteinuria and use of medication (hematinics, folic acid and herbal medication) (Table 6).

**Table 6:** Multivariate regression analysis for factors associated with anemia.

Variables	aOR (95%CI)	p-value
**Age (years)**		
<20	1	
20–30	1.13 (0.58–2.22)	0.723
>30	0.93 (0.43–2.01)	0.861
**Marital status**		
Single	1	
Married	0.64 (0.42–0.99)	0.044
**Source of drinking water**		
Purified water	1	
Tap water	3.05 (2.06–4.51)	<0.0001
Well water	2.45 (1.35–4.44)	0.003
**Obstetric parameters**		
**Gestational age**		
First trimester	1	
Second trimester	2.05 (1.41–2.97)	<0.0001
Third trimester	7.20 (3.06–16.92)	<0.0001
**Parity**		
0	1	
1	0.71 (0.43–1.16)	0.173
2	0.66 (0.37–1.20)	0.178
≥3	1.03 (0.59–1.82)	0.908
**Malaria**		
Absent	1	
Present	2.40 (1.27–4.53)	0.007

## Discussion

This study aimed to evaluate the prevalence and risk factors of malaria and anemia from three major hospitals across three regions in Ghana. The overall prevalence of malaria among the study population was 8.9%, with prevalence in the Ashanti, Greater Accra and Western regions being 5.5%, 10.1% and 11.4%, respectively. Self-reported exposure to mosquitoes and non-use of mosquito repellents were identified as a major risk factor for malaria in pregnancy; whereas increasing maternal age and parity reduced the risk of malaria. On the other hand, the prevalence of anemia was 54.5%, 34.3% and 37.1% in the Ashanti, Greater Accra and Western regions, respectively. Overall, the prevalence of anemia was 42.4%, with 35.7%, 6.1% and 0.6% presenting with mild, moderate and severe anemia, respectively. The use of unpurified/groundwater, malaria and gestational age were identified as major risk factors for anemia in pregnancy. Married pregnant women were less likely to develop anemia in pregnancy.

Our reported malaria prevalence of 8.9% is different compared to other studies in the country. In the Greater Accra region of Ghana, Stephens et al., reported 5.0% prevalence of *P*. *falciparum* parasitemia among pregnant women seeking antenatal care [30]. Also in the Greater Accra region, Ofori et al., found a 19.7% prevalence of malaria among pregnant women [26]. In the Ashanti region of Ghana, Tay et al., [31] reported 12.6% malaria prevalence among pregnant women. Another study in the Ashanti region by Darko et al., reported a 19.0% prevalence of malaria among pregnant women. In the Western region of Ghana, Orish et al. found 26.1% of the pregnant women to be infected with malaria parasites [27]. Clerk et al. [23] found a 47% prevalence of malaria parasitemia during pregnancy whereas Anabire et al., [32] observed a 15.5% prevalence of malaria both in northern Ghana. Thus, the evidence suggests intra- and inter-regional variations in the reported malaria prevalence rates. These discrepancies could be due the differences in the test method used, the effect of geographical location and seasonal variations on malaria endemicity. For instance, malaria is known to be prevalent in mid- and coastal regions of the country possibly due to the low patronage of the ITN by the people in the regions [21]. Furthermore, whereas most studies rely on malaria microscopy as a detection method, some use both microscopy and rapid diagnostic tests [27] and PCR [32]. Of note, the prevalence of malaria infection in this study was lower than the national figure of ~18% [33]. This could be due to the recent advances made in vector management strategies through free distribution of ITN throughout the country. Thus, intensifying efforts to make ITN more available and ensuring their proper use will be key to malaria prevention in the country.

In assessing the risk factors of malaria in pregnancy, we found age, marital status, source of drinking water, self-reported exposure to mosquitoes, parity, use of ITN and mosquito repellents to be possible contributors. After adjusting for potential confounding factors in multivariate analysis, we observed that, compared to younger pregnant women, older women (20–30 years old) had reduced odds of malaria infection. This finding is in harmony with studies by Clerk et al. [23] in Ghana, van Eijk et al., [34] in Kenya and Dicko et al., [35] in Mali. This could be related to the buildup of immunity to malaria over time through continuous exposure [36] particularly in endemic areas. Also consistent with previous reports [26, 31, 34], malaria prevalence decreased with increasing parity. Excessive exposure of mosquitoes increases the chance of mosquito bites; hence the risk of malaria. As expected, self-reported exposure to mosquitoes was an independent risk factor for malaria infection. Thus, it is important for pregnant women, a group with a high risk of malaria-related complications in pregnancy; to limit the rate of exposure to mosquito bites through the use of ITN or mosquito repellents. Indeed, as evidenced in this study, pregnant women who did not frequently use mosquito repellents presented higher odds of malaria infection. Notably, among those who used mosquito repellents, mosquito spray was predominantly employed. Indeed, the WHO recommends indoor residual spraying (IRS) with insecticides and use of ITN. IRS and ITN remains some of the powerful tools to rapidly reduce malaria transmission in the presence of high level of coverage [1]. Contrarily, we found no significant association between malaria and ITN use in this study; however, most of the pregnant women did not use ITN. This reinforces the need for the continuous and consistent distribution and ownership of ITNs to be encouraged and strengthened, particularly among such vulnerable groups as pregnant women.

Anemia in pregnancy still is a major public health problem and it is associated with maternal death, still births, low birth weights and impairment of fetal development [13, 17]. Our finding of a 42.4% prevalence of anemia among pregnant women is in keeping with the global estimate of more than 40% [10], the prevalence of anemia among pregnant women in this study was 42.4%. This finding is similar to the 40.8–54.0% prevalence reported in Ghana [25, 37, 38], 40.0% in Ethiopia [39], 40.1% in Mali [35], 42.7% in South Africa [40] and 47.4% in Tanzania [41]. Importantly, the prevalence of mild, moderate and severe anemia were 35.7%, 6.1% and 0.6%, respectively. The low prevalence of severe anemia is consistent with earlier reports [23, 24]. In pregnancy, anemia is linked to increased risk of maternal and neonatal mortality, preterm birth and low birth weight. Given the high prevalence of anemia in this study and the fact that pregnancy-related anemia is predominantly the synergistic effect of nutrition and economic status, there is the need to empower women economically and with interventions that reduce malnutrition through micronutrient supplementation and promotion of iron-rich products should be encouraged. The causes of anemia in pregnancy is complex and several predisposing factors have been implicated.

In this study, we found an association between anemia and marital status, source of drinking water, gestational age and parity. In the multivariate analysis, being married was associated with lower odds of anemia in pregnancy. This finding could be due to the important roles played by spouses during pregnancy. Studies in Ghana and Uganda have shown that, apart from the economic contribution, husbands play key roles in the frequency of a pregnant spouse’s ANC visits, with some men taking up the responsibility of ensuring that their wives attend ANC clinics and conforms to the directives of their physician/nurse. Thus ensuring the overall well-being of their pregnant spouses and thus contributing to reducing anemia in pregnancy [42, 43]. We thus recommend community-level health education that enhances involvement of men and family members during pregnancy for maximum impact in the fight against anemia in pregnancy.

Interestingly, controlling for confounders, women who drank groundwater/unpurified water presented with over two-fold increased risk of anemia compared to the drinking purified water. Groundwater is used by over 90% of the Ghanaian population for several purposes. Evidence suggests some level of dissolved iron in groundwater which can improved the iron status [44]. Antithetically, current data indicate that drinking groundwater increases the likelihood of anemia [45]. This is because groundwater has been shown to contain high concentrations of arsenic [46]. Arsenic leads to anemia through several mechanisms including decreasing heme metabolism, lowering hemoglobin concentrations by binding to hemoglobin, altering erythrocyte morphology and inducing erythrocyte death, and depressing bone marrow hematopoiesis [47–49]. Consistent with our finding, Hopenhayn et al., [50] in Chile found that, after adjusting for confounders, women exposed to arsenic through groundwater were more likely to be anemic during pregnancy. Moreover, as pregnancy progressed, the prevalence of anemia rose more sharply among those exposed to arsenic compared to the unexposed. Ghana has made substantial progress toward the Millennium Development Goals’ target of making safe drinking water accessible to its population; however, a substantial number of the people, particularly those in marginalized regions, still lack potable drinking water [51]. Adopting an integrated approach to bridge the marginalized–urban gap with respect to access to improved drinking water is recommended.

Another finding of this study was that increasing gestational age was independently associated with increased odds of anemia in pregnancy. This finding is similar to a study in Ghana by Dicko et al., [35] who found that the risk of anemia was lower during the first trimester of pregnancy. Clerk et al., [23] in Ghana and Gedefaw et al., [39] in Ethiopia also made similar observations and our finding partly corroborates the study by Mockenhaupt et al., [37] among Ghanaian pregnant women. This finding could be due the reduction in maternal iron reserves with increasing gestational age as a result of increasing demand by the fetus. Similarly, Yuan et al., [52] found a high prevalence of iron deficiency in late pregnancy and reported its associations with birth outcomes in Chinese pregnant women. In Ghana, interventions such as free provision of ITNs, health and nutrition education, screening and treatment of anemia and other conditions and provision of multivitamins and mineral supplements occur during ANC visits. However, as evidenced from this study, over 50% of the pregnant women had their first ANC visit at the second/third trimester. The delay in first ANC visit reduces the chance of early identification and treatment of anemia or any other related condition. Indeed, women who had their first ANC visit at the second trimester had a two-fold increase in the risk of anemia whereas the risk was seven-fold for those in the third trimester. We recommend intensified education of women on early initiation of antenatal care to enhance surveillance, identification and treatment of anemia.

Importantly, among the pregnant women with anemia, 5.1% of them had malaria. Regression analysis from our study, revealed that malaria increases the risk of anemia by two-folds among the population. Other studies have also reported increased risk of anemia with malaria among pregnant women in Ghana and elsewhere [35, 37, 53–55] This finding could be linked to the intricate relationship between malaria and anemia such as malaria-induced destruction of non-parasitized and parasitized red cells, impaired erythropoiesis and delayed reticulocyte response due to suppression of the normal erythropoietin response during periods of tissue hypoxia [14–16]. Nonetheless, it is worthy of note that, malaria contributed only 4.5%, 0.3% and 0.3% to the prevalence of mild, moderate and severe anemia, respectively, suggesting that the presence of other factors, as indicated in this study, also play important roles in anemia in pregnancy. This notwithstanding, increasing effort through public health education on the importance and use of malaria preventive strategies such as use of ITNs and IRS could help combat the increasing prevalence of both anemia and malaria in Ghana.

### Strengths and limitations

The strength of this study lies in establishment of multiregional prevalence of malaria and anemia among pregnant women in the middle and lower belt of Ghana along with presenting reliable data on the risk factors of malaria and anemia among pregnant women in Ghana. However, some limitations need to be acknowledged. First, although the data was obtained from three major hospitals which serves three regions of the country; hence could represent the current state in those regions, data from the northern belt is not provided in this study; thus, the findings is not to be generalized as data for the whole country. Further studies including the northern sector of the country is warranted. Additionally, although microscopy remains the standard for malaria diagnosis in Africa, the test method has been shown to be less sensitive. Thus, it is possible that the prevalence of malaria may have been underestimated due to missed cases. Future studies should consider the use of more sensitive diagnostic methods like polymerase chain reaction. This study was also conducted using a convenient sampling method.

## Conclusion

Although the prevalence of malaria is low (8.9%), that of anemia remains high (42.4%) among pregnant women in Ghana. The major risk factors of malaria among pregnant women in Ghana are exposure to mosquitoes and non-use of mosquito repellents whereas the risk factors for anemia in pregnancy are use of unpurified/groundwater and gestational age. Increasing maternal age and parity reduces the risk of malaria whiles married pregnant women have lower risk of anemia in pregnancy. Malaria is a significant contributor to anemia in pregnancy; however, other factors are similarly important. We recommend increasing efforts to make ITNs more available to strengthen malaria prevention. Public health education programs could help improve uptake and proper use of ITNs. To help reduce anemia in pregnancy, women should be empowered economically and interventions that reduce malnutrition through micronutrient supplementation and promotion of iron-rich products should be encouraged. Additionally, community-level health education that enhances involvement of men and family members during pregnancy as well as adopting an integrated approach to bridge the rural–urban divide with respect to access to improved drinking water is recommended. We also recommend educating women on early initiation of antenatal care to enhance surveillance, identification and treatment of anemia.

## Supporting information

S1 File Questionnaire. (DOCX)
