# Supplementary material for: A multicenter study of the prevalence and risk factors of malaria and anemia among pregnant women at first antenatal care visit in Ghana
Source: PLoS One. 2020 Aug 21;15(8):e0238077. doi: 10.1371/journal.pone.0238077 (PMC7444479; doi:10.1371/journal.pone.0238077)
Supplement: S1 File — (DOCX) [file pone.0238077.s001.docx]

**QUESTIONNAIRE ON MALARIA AND ANAEMIA IN PREGNANT WOMEN AT FIRST ANTENATAL CLINIC VISIT IN GHANA**

Date: _____/_____/__________

**SOCIO-DEMOGRAPHICS**

|  |  |  |
| --- | --- | --- |

**Participant number**

**Name** …………………………………………………………………………………………

|  |  |  |  |  |  |  |  |
| --- | --- | --- | --- | --- | --- | --- | --- |

**Date of birth (*dd/mm/yyyy*)**

**Ethnicity**……………………………………………………………………………………….

**Place of residence** ………………………….....................................................................

**Relationship status:**

*Single*

*Married*

*Divorced*

*Separated*

*Widowed*

*Cohabiting*

**Educational background:**

*Never*

*Primary school*

*JHS*

*SHS*

*Post-sec training*

*Tertiary*

**Occupation:**

*Informal*

*Formal skilled*

*House wife*

*Student*

*Not employed*

*Other (please specify)* ……....

**MATERNAL HEALTH**

**How many pregnancies have you had (gravidity)?** ………………………………………………..

**How many previous deliveries have you had (parity)?** …………………………………………

**How old is this pregnancy (gestational age)?**  ..……… *month(s)* ………. *week(s)*

**Have you been treated or hospitalized for malaria recently?** *Yes*  *No*

**If yes, how many weeks has it been since then?** ………. *week(s) ……….day(s)*

**Have you been diagnosed or treated for anaemia recently?** *Yes*  *No*

**Are you on any of these medications?**

*Antimalarials*

*Folic acid*

*Haematinics*

*Herbal preparations*

*Antipyretics*

*Anthelminthics*

**Have you had fever the past week?** *Yes*  *No*

**Have you been diagnosed of any chronic disease?** If yes, please specify ………………………………….

**QUALITY OF LIVING**

**On the scale of 1 to 10, how severely have you been exposed to mosquito bites within the past month? ***................................................**

**Do you use an Insecticide Treated Net?** *Yes*   *No*

**Do you use an Insecticide Treated Net on a daily basis?** *Yes*   *No*

**If yes, how often do you treat your net?**

*Never*

*Every 3 months*

*Every 6 months*

*Other (please specify)*

**Do you use any of these mosquito repellents?**

*Mosquito coils*

*Insecticide sprays*

*Other repellents (please specify)*

**Do you have access to electricity?** *Yes*  *No*

**What is you main source of drinking water:**

*Tap water*

*Purified water*

*Well water*

*Other (please specify)*…………………..

**What is your home type?**

*Self-owned house*  *Compound/shared house*

**Number of persons in your household:** ……………………………………

**FAMILY HISTORY**

**Do you have any family history of?**

Sickle Cell Disease *Yes*  *No*  *Not sure*

G6PD Deficiency *Yes*  *No*  *Not sure*

Renal Disease *Yes*  *No*  *Not sure*

***Notes:***

******* Grading for exposure to mosquito bites: *Barely=1-3, moderate=4-7, severe=8-10***
